# Right Internal Jugular Vein Phlebectasia: A Rare Cause of Neck Swelling

**DOI:** 10.1155/2017/9278728

**Published:** 2017-12-18

**Authors:** Deepanjan Bhattacharya, Mounika Endrakanti, Rakesh Kumar

**Affiliations:** Department of Paediatrics, PGIMER, Chandigarh, India

## Abstract

Internal jugular vein (IJV) phlebectasia is a rare condition presenting as a self-reducible soft tissue swelling of the neck due to fusiform dilation of the venous wall. We report the case of a 7-year-old boy who presented with painless soft mass in the side of the neck which appears during coughing and straining and reduces spontaneously. Diagnosis was confirmed by Doppler ultrasonography and magnetic resonance imaging of the neck. Conservative management and regular follow-up were considered. In this case report, we highlight this rare benign condition as an uncommon differential of neck swellings in order to avoid unnecessary diagnostic workup and interventions.

## 1. Introduction

Phlebectasia is an abnormal fusiform dilation of a vein. It is a rare venous malformation, and etiology is not clear. It occurs more commonly in the upper extremity and cervical veins and tends to be asymptomatic without serious consequences as opposed to lower limb venous aneurysms which may complicate with pulmonary thromboembolism in adults. Phlebectasia differs from venous aneurysms, which are usually secondarily acquired, segmental, saccular, and typically seen in adults. It also differs from varices which are tortuous dilations of the veins.

## 2. Case Report

**Figure 1:**
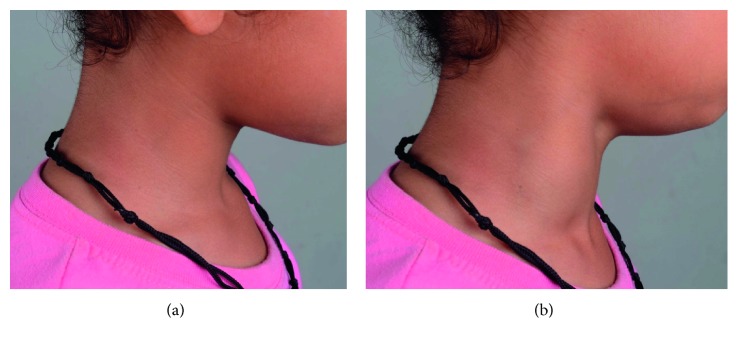
Photographs showing appearance of swelling in the neck: (a) when patient is not coughing and (b) when patient is coughing.

**Figure 2:**
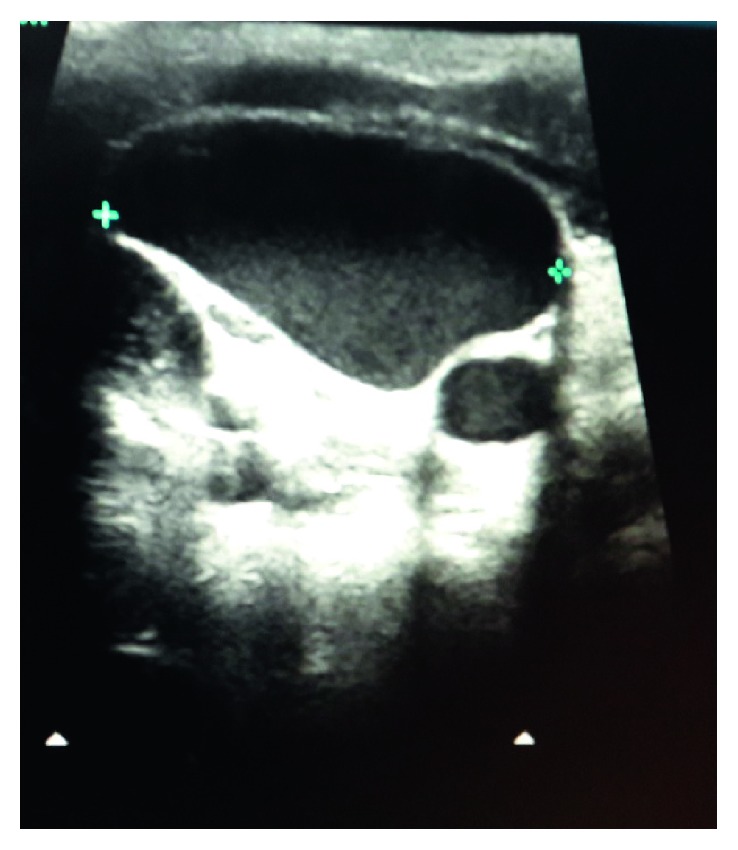
Grayscale ultrasound of the neck reveals fusiform dilatation of the right internal jugular vein.

**Figure 3:**
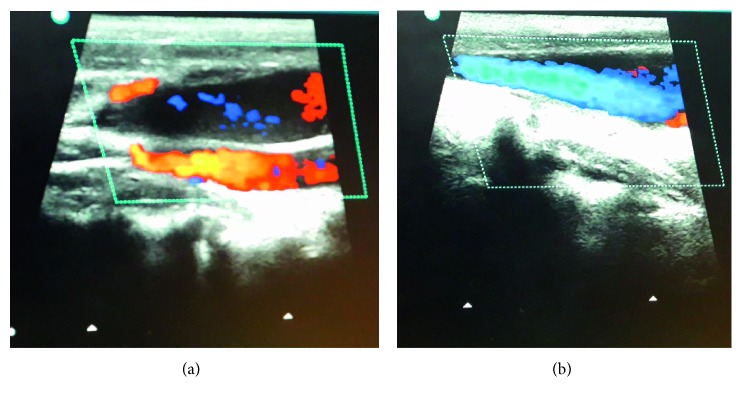
(a, b) Colour Doppler study of the neck showing uniform colour flow in the dilated internal jugular vein with no thrombosis.

**Figure 4:**
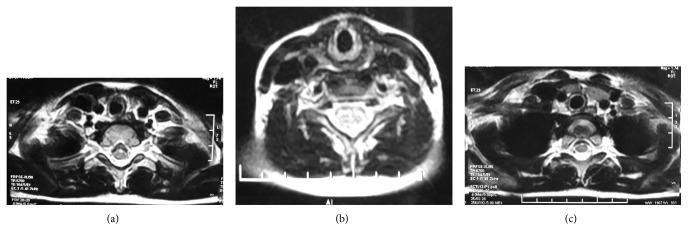
(a, b, and c) Axial T2-weighted MRI of the neck reveals dilatation of the right internal jugular vein.

A 7-year-old otherwise healthy boy presented to outpatient department with a complaint of a painless lump appearing on the right side of the neck while coughing, noticed for the past 3 days. It was not associated with fever, coryza, dyspnea, dysphagia, dysphonia, or facial congestion. There was no history of pain or antecedent trauma or surgery. Family history was unremarkable. On examination, his anthropometric measurements were within the normal range. He had 3 × 3 cm, nontender fusiform swelling in the right side of the neck, anterior to the sternocleidomastoid muscle, appearing only at the time of coughing, extending to the anterior triangle of the neck, and the clavicle (Figure 1). There was no palpable thrill or audible bruit over the swelling. No lymph nodes were palpable in the neck, and oropharyngeal examination was unremarkable. Systemic examination did not reveal any abnormality. USG of the neck showed fusiform dilatation of the lower segment of the IJV with a caliber of 2.5 cm, increasing on coughing to 3.1 cm (Figure 2). The Doppler study revealed turbulence in the IJV without any evidence of thrombosis (Figure 3). MRI of the neck demonstrated fusiform dilatation of the lower segment of the IJV, increasing in size during coughing (Figure 4). Parents were counseled about the benign nature of the condition. Vascular surgery consultation was sought, and conservative management with regular follow-up was planned in view of absence of secondary complications.

## 3. Discussion

Phlebectasia indicates outward dilatation of the vein without tortuosity and thus is different from the varix. Zukschwerdt first reported this condition in 1929 and was subsequently described by Gerwig [1]. The etiology is not clear; histopathology is mostly normal; few have reported disarray of the smooth muscle, elastic fibres, and connective tissue [2]. LaMonte hypothesized that since the right innominate vein lies in close contact with the right apical pleura, any increase in intrathoracic pressure would increase the pressure on the IJV, making phlebectasia more common on the right side [3].

Jugular venous phlebectasia has been more commonly described in pediatric population, presenting as a soft compressible swelling in the neck and increasing in size during coughing, crying, sneezing, or Valsalva maneuver. It has been reported to be found in all neck veins, though most commonly occurring in the IJV, followed by the external jugular vein, anterior jugular vein, and other communicating veins. It is twice more common in boys as compared to girls and usually occurs on the right side [4]. The etiology of this disease is unclear and is attributed to anatomic abnormality, mechanical compression, trauma, and congenital defects [5]. It is usually asymptomatic but can cause hoarseness of voice or Horner's syndrome and can be complicated by thrombosis. It may also have intracranial extension without any features suggestive of CNS involvement [6].

It is a relatively benign condition, and conservative management is the norm. Surgical treatment is indicated in case of complications like phlebitis, thrombosis, and Horner's syndrome or for cosmetic reasons. Surgical options include ligation of the jugular vein, excision of the dilated segment [7], and sheathing of the affected segment in a polytetrafluoroethylene graft. However, ligation has been attributed to venous oedema of the brain, and resection and sheathing are favoured.

This case report highlights phlebectasia of the IJV, a rare benign condition as a differential of neck swelling. Enlargement of the neck mass on Valsalva maneuver observed in this condition limits the differential diagnoses to laryngocele and superior mediastinal cyst or tumor, branchial cyst, cystic hygroma, and cavernous haemangioma. The Doppler study is useful to differentiate it from laryngocele. Since this condition is an extreme rarity, one must keep high index of suspicion to diagnose it and avoid unnecessary interventions.
